# Hepatic Proteomic Changes and Sirt1/AMPK Signaling Activation by Oxymatrine Treatment in Rats With Non-alcoholic Steatosis

**DOI:** 10.3389/fphar.2020.00216

**Published:** 2020-03-10

**Authors:** Hong Xu, Gao-Feng Chen, Yu-Shui Ma, Hong-Wei Zhang, Yang Zhou, Guang-Hui Liu, Dong-Ya Chen, Jian Ping, Yi-Hui Liu, Xin Mou, Da Fu

**Affiliations:** ^1^Department of Gastroenterology and Hepatology, Hangzhou Red Cross Hospital, Hangzhou, China; ^2^Institute of Liver Diseases, Shuguang Hospital Affiliated to Shanghai University of Traditional Chinese Medicine, Shanghai, China; ^3^Central Laboratory for Medical Research, Shanghai Tenth People’s Hospital, Tongji University School of Medicine, Shanghai, China; ^4^Shanghai Institute for Advanced Immunochemical Studies, ShanghaiTech University, Shanghai, China; ^5^Liver Cirrhosis Section, Department of Hepatology, Shuguang Hospital Affiliated to Shanghai University of Traditional Chinese Medicine, Shanghai, China; ^6^Department of Endocrinology, Tongji Hospital, Tongji University School of Medicine, Shanghai, China; ^7^Department of Endocrinology, Hangzhou Red Cross Hospital, Hangzhou, China

**Keywords:** NAFLD, proteomic, iTRAQ, oxymatrine, sirtuin 1, AMPK

## Abstract

**Background:**

Currently, active ingredients of herbal extracts that can suppress lipid accumulation in the liver have been considered a potential treatment option for non-alcoholic fatty liver disease.

**Methods:**

Steatosis rat model was created by high fat and high sucrose diet feeding and treated with oxymatrine (OMT). Serum biochemical parameters, liver histology and lipid profiles were examined. Hepatic differentially expressed proteins (DEPs) which were significantly changed by OMT treatment were identified by iTRAQ analysis. The expressions of representative DEPs, Sirt1 and AMPKα were evaluated by western blotting.

**Results:**

OMT significantly reduced the body weight and liver weight of steatosis animals, decreased the serum levels of triglyceride and total cholesterol as well as the hepatic triglyceride and free fatty acid levels, and effectively alleviated fatty degeneration in the liver. A list of OMT-related DEPs have been screened and evaluated by bioinformatics analysis. OMT significantly decreased the expressions of L-FABP, Plin2, FASN and SCD1 and increased Sirt1 expression and AMPKα phosphorylation in the liver of rats with steatosis.

**Conclusion:**

The present study has confirmed the significant efficacy of OMT for improving steatosis and revealed hepatic proteomic changes and Sirt1/AMPK signaling activation by OMT treatment in rats with steatosis.

## Introduction

Non-alcoholic fatty liver disease (NAFLD), an important public health issue worldwide, includes a spectrum of diseases ranging from simple steatosis to a more aggressive condition of steatohepatitis. Steatohepatitis is characterized by steatosis, inflammation, and hepatocyte injury and may progress to cirrhosis. The high prevalence of NAFLD is closely associated with the dramatic rise in obesity, diabetes mellitus and dyslipidemia. It has been reported that NAFLD affects approximately 60–90% of obese individuals, up to 70% of diabetics and 27–92% of patients with hyperlipidemia (Pappachan et al., 2017; Younossi et al., 2018). NAFLD is currently considered an independent predictor of long-time adverse cardiovascular events (Wu et al., 2016). Thus, identifying potential therapeutic targets and developing new drugs for NAFLD would have substantial clinical value.

The development of NAFLD is a complex multi-factorial process with strong genetic, environmental and metabolic contributions. The traditional “two-hit” hypothesis, which is widely accepted as a framework for guiding researches in the area, states that insulin resistance and increased FFA contribute to excessive lipid accumulation in hepatocytes in the first hit, while the second hit represents increased oxidative stress initiating lipid peroxidation and inflammatory insult to the liver (Basaranoglu et al., 2013). Recently, the “multiple-hit” hypothesis has been proposed as the more logical mechanism (Buzzetti et al., 2016).

Although comprehensive studies have been carried out to investigate the complicated molecular mechanism of NAFLD, its pathogenesis and progression still remain elusive. Proteomics is a powerful tool for the identification of novel biomarkers and potential therapeutic targets for NAFLD. iTRAQ has been widely applied in comparative proteomics due to the advantages of high throughput, high sensitivity and superior accuracy over conventional approaches (Unwin et al., 2010; Lim et al., 2014). Currently, increasing efforts have been made to identify active ingredients of herbal extracts, which have minimal side effects and aim at multiple targets, to expand the treatment options for NAFLD (Hsu et al., 2016).

Oxymatrine (OMT), a potent monosomic alkaloid derived from the root of *Sophora flavescens Ait*, has been reported to possess anti-inflammatory, anti-oxidative and hepatoprotective activities (Lu et al., 2016; Zhao et al., 2016; Li et al., 2017). Available studies revealed that OMT attenuated hepatic steatosis through the down-regulation of sterol regulatory element binding transcription factor 1 (SREBF1) and up-regulation of peroxisome proliferator activated receptor alpha (PPARα) mediated metabolic pathway (Shi L.J. et al., 2013). However, these studies only superficially detected the mRNA and protein expressions of SREBF1 and PPARα. The underlying mechanism of OMT on improving steatosis is incompletely understood.

Therefore, in the present study, a rat model of hepatic steatosis was created by high fat and high sucrose diet (HFHSD) feeding and treated with OMT. The aim of our study was to identify a list of DEPs in the liver by iTRAQ-based proteomic analysis to unveil the potential therapeutic targets of OMT for improving steatosis.

## Materials and Methods

### Reagents and Equipments

Oxymatrine (purity >98%) was purchased from Sigma-Aldrich. Hepatic TG and FFA assay kits were from Nanjing Jiancheng Bioengineering Institute. Sequencing-grade trypsin was from Promega. iTRAQ reagent-8 plex multiplex kit was obtained from Applied Biosystems. Triple TOF 5600 mass spectrometer and Eksigent nanoLC-1D plus liquid chromatography were from SCIEX. Rabbit anti-Plin2, rabbit anti-L-FABP, rabbit anti-FASN, rabbit anti-Sirt1 and mouse anti-SCD1 monoclonal antibodies were from Abcam. Rabbit anti-AMPKα (phospho-Thr172) and rabbit anti-AMPKα monoclonal antibodies were from Cell Signaling Technology.

### Animals, Ethics Statement and Treatment Protocol

Male Sprague-Dawley rats were obtained from Sino-British SIPPR/BK Lab Animal Ltd (SCXK [Shanghai] 2013-0016) and reared in the specific pathogen free facility of the Experimental Animal Research Center, Zhejiang Chinese Medical University. All animals received humane care with strict accordance to the criteria outlined in the Guide for the Care and Use of Laboratory Animals. The study was reviewed and approved by the Committee on Animal Research and Ethics of Zhejiang Chinese Medical University (number ZSLL-2014-36).

Steatosis was induced by HFHSD feeding for 8 weeks. Thirty rats weighing 180–200 g were equally randomized into three groups: control group (*n* = 10), model group (*n* = 10) and OMT-treated group (*n* = 10). The rats in the model group and OMT-treated group were fed with HFHSD, which consist of 74.25% standard chow, 10% sucrose, 0.5% cholesterol, 5% egg yolk powder, 10% lard, 0.25% sodium cholate. The rats in OMT-treated group additionally received intragastric administration of OMT at a dose of 100 mg/kg/day. The control rats were fed a standard chow and received normal saline intragastrically.

### Serum Biochemical Assays

The activities of serum ALT and AST and the levels of triglyceride (TG) and total cholesterol (TC) were determined by an Beckman Coulter AU5800 automatic biochemical analyzer.

### Liver Histological Examination

Liver specimens were fixed in 4% paraformaldehyde, dehydrated in a graded alcohol series and embedded in paraffin. Sections of 4 μm thickness were stained with hematoxylin and eosin (H&E). To determine hepatic lipid accumulation, fresh liver tissues were embedded in OCT compound and frozen in liquid nitrogen. Frozen sections (10 μm) were stained with Oil Red O. The percentage of Oil Red-positive staining area was calculated by using ImagePro Plus software from five to seven views per animal.

### Hepatic Lipid Profiles

The levels of TG and FFA in the liver were determined using commercial kits according to the manufacturer’s protocols.

### Protein Extraction, Digestion, and iTRAQ Labeling

Frozen liver tissues were ground into powder using liquid nitrogen. The powder was homogenated in ice-cold SDS lysis buffer (containing 50 mM Tris, 0.1% SDS, 1% Triton X-100 and protease inhibitor cocktail). The lysate was centrifuged at 12,000 *g*, 4°C for 15 min. The supernatant was collected and precipitated with ice-cold acetone (1:5, v/v) at −20°C overnight. The pellet was collected by centrifugation, washed twice with ice-cold acetone, dried and resolved with sample buffer (7 M urea, 2 M thiourea, 50 mM DTT, 1 mM PMSF, 50 mM Tris, 1 mM RNAse, and 1 mM DNAse). Protein concentration was determined and equal amount of total protein from several rats in the same group were pooled together as a biological replicate to alleviate the individual variability. Two or three biological replicates were acquired.

A total of 200 μg of pooled proteins from each group was reduced, alkylated, and digested by sequencing-grade trypsin. Briefly, the samples were was incubated with 200 μl reducing buffer (10 mM DTT, 8M urea, 100 mM TEAB, 150 mM Tris–HCl, pH 8.0) for 1 h at 60°C, cooled to room temperature, alkylated with 50 mM iodoacetamide (IAA) for 1 h in darkness and subjected to 10 kDa ultrafiltration. The sediment were collected and digested by sequencing-grade trypsin at 37°C for 16 h, with ratios of protein to trypsin of 50:1, and labeled using iTRAQ reagent-8 plex Multiplex Kit according to the manufacturer’s protocol (Liu X. et al., 2017).

### Reversed Phase Liquid Chromatography Fractionation

The iTRAQ-labeled peptide mixtures were separated using an Agilent Zorbax Extend RP column (C18, 5 μm, 150 mm × 2.1 mm). Mobile phases A (2% ACN in water) and B (98% ACN in water) were used for gradient. The solvent gradient was set as described previously (Hu et al., 2018). Peptides were separated at a fluent flow rate of 300 μL/min and monitored at 210 nm and 280 nm. Dried samples were harvested from 8 to 50 min and elution buffer were collected in every minute and numbered from 1 to 10 with pipeline. The separated peptides were lyophilized for MS detection.

### LC-MS/MS Analysis

All analyses were performed by a Triple TOF 5600 mass spectrometer equipped with a Nanospray III source. Samples were separated by a reverse-phase C18 column (15 cm × 75 μm, 3 μm, 120 Å) on an Eksigent nanoLC-1D plus system. Mobile phase *A* = 2% ACN/0.1% FA and *B* = 95% ACN/0.1% FA. The flow rate was 300 nL/min and linear gradient was set as described previously (Hu et al., 2018).

Data were acquired with a 2.4 kV ion spray voltage, 35 psi curtain gas, 5 psi nebulizer gas, and an interface heater temperature of 150°C. The MS scanned between 400 and 1500 with an accumulation time of 250 ms. For IDA, 30 MS/MS spectra (80 ms each, mass range 100–1500) of MS peaks above intensity 260 and having a charge state of between 2 and 5 were acquired. A rolling collision energy voltage was used for CID fragmentation for MS/MS spectra acquisitions. Mass was dynamically excluded for 22 s (Yu et al., 2019).

### Database Search

The original MS/MS file data were analyzed by ProteinPilot Software v5.0. Processing parameters were set as follows: iTRAQ 8-plex quantification, cysteine modified with IAA; biological modifications were selected as ID focus, trypsin digestion; protein quantification and normalization were checked by the Background Correction, Quantitate and Bias Correction (Yao et al., 2018). Proteins with at least 95% confidence determined by Protein Pilot Unused scores (≥1.3) were reported, and the FDR was set up less than 1%. Fold changes ≥1.3 were considered significant.

### Bioinformatics Analysis

The interaction networks of DEPs were analyzed by STRING database^1^. The BP, CC, and MF were analyzed by GO database^2^. We defined the significance of GO enrichment according to a *P* value <0.05. The pathway analysis was performed by KEGG database^3^.

### Western Blot Analysis

Samples were prepared as described previously (Xu et al., 2016). Equal amounts (30–50 μg) of protein were loaded on 8% or 12% SDS-polyacrylamide gel, separated and transferred to polyvinylidene difluoride membranes. The blots were probed with rabbit monoclonal antibodies against Plin2 (1:1000), L-FABP (1:1000), FASN (1:1000), Sirt1 (1:1000), AMPKα (1:1000), AMPKα (Thr172) (1:1000) and mouse monoclonal antibody against SCD1 (1:1000) followed by HRP-conjugated goat anti-rabbit IgG or rabbit anti-mouse IgG. Signals were visualized by enhanced chemiluminescence detection.

### Statistical Analysis

Statistical Package for the Social Sciences (SPSS version 20.0) software was used for the statistical analysis. Data are presented as mean ± standard deviation (SD) and analyzed by one-way ANOVA. Bonferroni’s correction was performed to adjust for multiple comparisons. *P* < 0.05 was considered as statistically significant.

## Results

### OMT Reduces Body Weight and Liver Weight in Steatosis Rats

**FIGURE 1:**
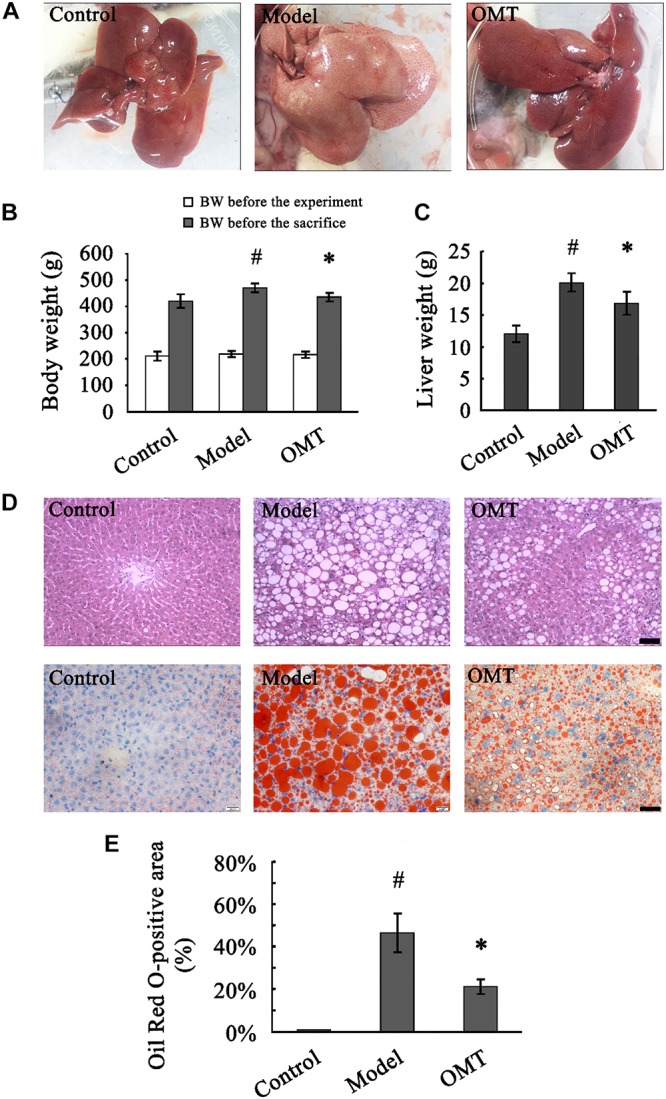
OMT reduces body weight and liver weight and alleviates hepatic fatty degeneration in rats with steatosis. **(A)** Macroscopic evaluation of the livers. **(B)** Body weights of the animals before the experiment and before the sacrifice. #*P* < 0.01 vs. the control group, ^∗^*P* < 0.01 vs. the model group. **(C)** Liver weight. #*P* < 0.01 vs. the control group, ^∗^*P* < 0.01 vs. the model group. **(D)** Histological evaluation of liver tissues by H&E and Oil Red O staining. Scale bar, 30 μm. **(E)** Quantitative analysis of liver steatosis by Oil Red O staining. The percentage of Oil Red O-positive staining area was calculated according to the following formula: Oil Red O-positive area/total area × 100%. #*P* < 0.001 vs. the control group, ^∗^*P* < 0.001 vs. the model group.

Prior to the experiment, no differences were observed in body weight among the three groups. After 8 weeks of HFHSD feeding, macroscopic appearances of the livers from the model rats were enlarged, yellowish and greasy, whereas the control rats displayed brown, smooth, and shiny liver tissues. Accordingly, the animals in the model group showed significantly increased body weight and liver weight compared to those in the control. The abnormal macroscopic appearance of the liver was significantly improved by OMT treatment. Furthermore, OMT effectively decreased the body weight as well as the liver weight (Figures 1A–C).

### OMT Alleviates Hepatic Fatty Degeneration

Representative images of randomly selected sections by H&E and Oil Red O staining were shown in Figure 1D. The control rats exhibited a normal liver architecture with well-arranged hepatic lobules. Severe fatty degeneration with destroyed structure of normal hepatic lobules was observed in the livers of the model rats. The percentage of Oil Red O-positive staining area in the model group was significantly higher than that in the control (46.50 ± 9.08% vs. 0.90 ± 0.13%). The fatty degeneration was noticeably improved by OMT treatment with the percentage of Oil Red O-positive staining area (21.15 ± 3.40%) significantly decreased compared to the model group (Figure 1E).

### OMT Improves Serum and Hepatic Lipid Profiles

**FIGURE 2:**
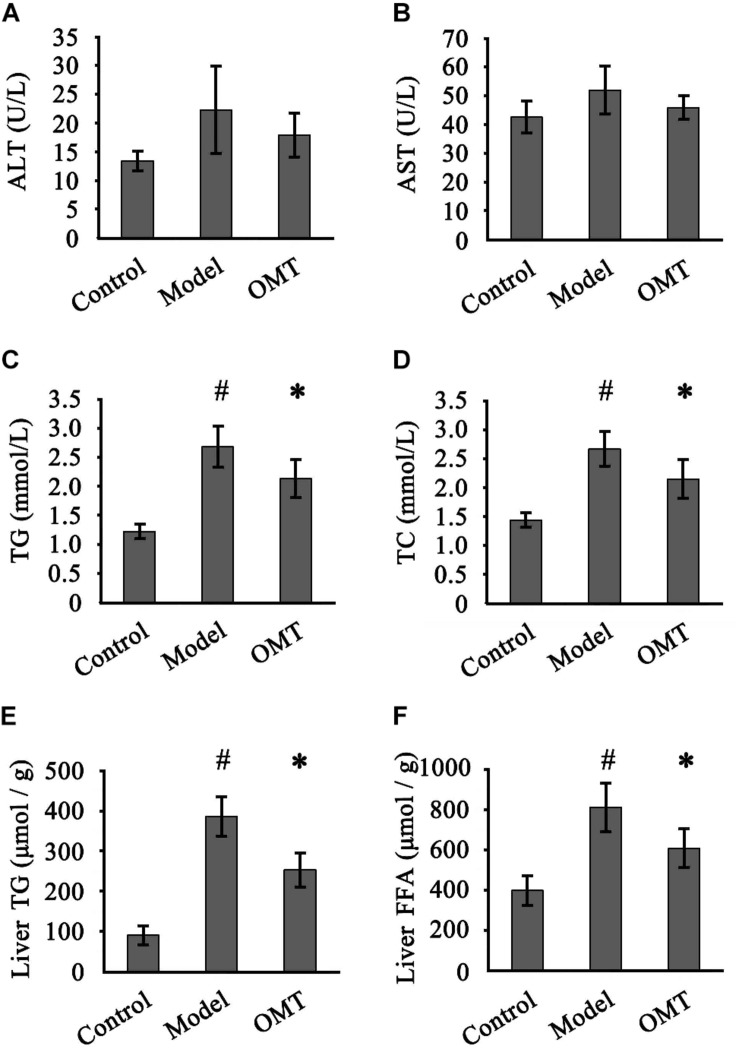
OMT improves serum and hepatic lipid profiles. Serum levels of ALT **(A)** and AST **(B)** among the three groups were detected. Serum levels of TG **(C)** and TC **(D)**. Hepatic levels of TG **(E)** and FFA **(F)**. Data are representative of 10 rats in each group and expressed as mean ± SD. #*P* < 0.01 vs. the control group, ^∗^*P* < 0.01 vs. the model group.

Slightly but not statistically increased serum ALT and AST levels were detected in the model group compared to the control and OMT-treated groups (Figures 2A,B). Compared to the control group, the levels of serum TG and TC in the model group were remarkably increased (2.68 ± 0.35 mmol/L vs. 1.22 ± 0.12 mmol/L and 2.67 ± 0.29 mmol/L vs. 1.44 ± 0.12 mmol/L, respectively). OMT treatment significantly reduced serum TG and TC levels to 2.13 ± 0.32 mmol/L and 2.15 ± 0.33 mmol/L, respectively (Figures 2C,D). Similarly, the model group displayed significantly higher hepatic TG and FFA levels than the control (386.2 ± 47.8 μmol/g vs. 90.8 ± 22.7 μmol/g and 812.0 ± 118.3 μmol/g vs. 398.4 ± 72.6 μmol/g, respectively). OMT significantly decreased hepatic TG and FFA levels (Figures 2E,F).

### iTRAQ Quantitative of DEPs

**FIGURE 3:**
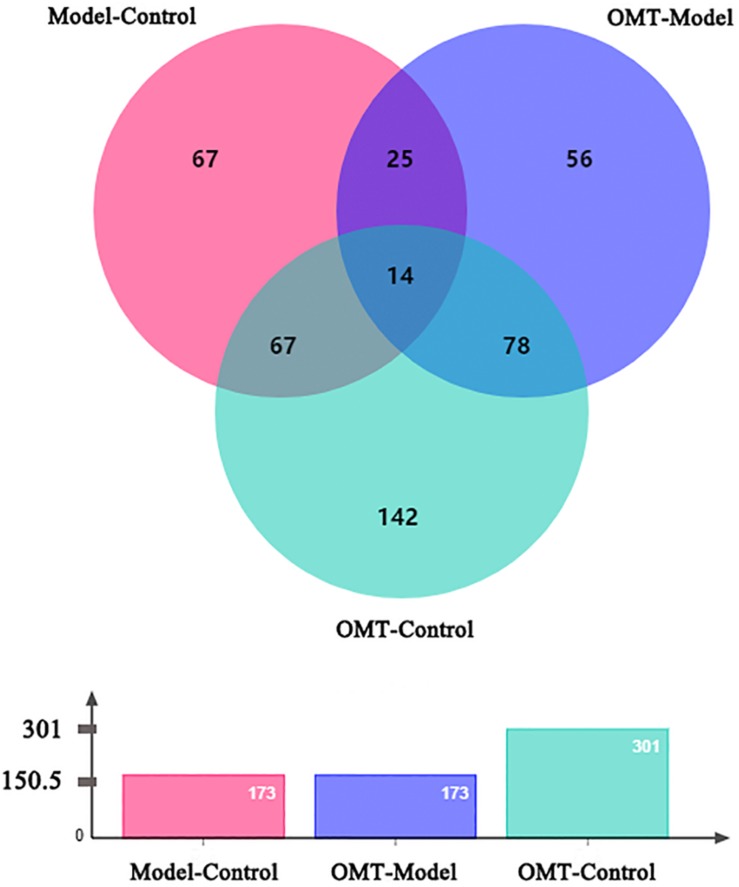
Venn-diagram depicts the overlap of all DEPs among the three groups.

iTRAQ-based proteomic analysis was used to detect DEPs in each group. After merging the data from two or three independent biological replicates, a total of 2859 proteins were identified and quantified against the Rat Database (FDR < 1% and containing at least two unique peptides). 173 DEPs (*p* < 0.05 and changes >1.3-fold) were identified between the model group and the control group, of which 77 proteins were up-regulated and 96 proteins were down-regulated. 173 DEPs were identified between the OMT-treated group and the model group, of which 88 proteins were up-regulated and 85 proteins were down-regulated. Furthermore, 301 differential proteins were identified between the OMT-treated group and the control group with 141 proteins up-regulated and 60 proteins down-regulated (Figure 3).

We classified all DEPs into two categories: steatosis-specific DEPs, which were up-regulated or down-regulated significantly in the liver of the model group compared to the control, and OMT-related DEPs. OMT-related DEPs include a bunch of steatosis-specific DEPs, which were regulated in an opposite way following OMT treatment. Representative proteins included perilipin 2 (Plin2), liver fatty acid-binding protein (L-FABP, also known as FABP1), FASN and stearoyl -CoA desaturase 1 (SCD1). The expressions of some proteins remained unchanged throughout HFHSD feeding, but significantly changed by the administration of OMT. After the removal of the proteins overlapped with steatosis-specific DEPs, there were 177 DEPs between the OMT-treated group and control group. These proteins were also defined as OMT-related DEPs.

Representative OMT-related DEPs were shown in Table 1. These proteins were mainly involved in fatty acid metabolism (Fasn, Scd1, Fads2, Fdps, Plin2), fatty acid degradation (Ehhadh, Acsl1), PPAR signaling pathway (Fads2, Ehhadh, Acsl1, Fabp1), non-alcoholic fatty liver disease (Ehhadh, Pklr) and AMPK signaling pathway (Fasn, Scd1, Hnf4a).

**TABLE 1:** Representative OMT-related DEPs detected by iTRAQ analysis.

**Protein name**	**Uniprot accession**	**Model/Control (fold)**	***P* value**	**OMT/Model (fold)**	***P* value**
Plin2	tr| Q5U2U5| Q5U2U5_RAT	2.80	2.044E-05	0.54	0.000
Fabp1	sp| P02692| FABP1_RAT	2.56	0.001	0.52	0.003
Fasn	sp| P12785| FAS_RAT	3.12	0.000	0.46	0.004
Scd1	sp| P07308| ACOD1_RAT	2.43	0.000	0.48	0.000
Fads2	sp| Q9Z122| FADS2_RAT	0.53	0.017	1.98	0.003
Hnf4a	tr| G3V750| G3V750_RAT	1.89	0.015	0.49	0.005
Pklr	sp| P12928| KPYR_RAT	2.23	0.007	0.54	0.006
Fdps	tr| F1LND7| F1LND7_RAT	0.35	0.000	1.97	0.001
Rab18	sp| Q5EB77| RAB18_RAT	2.16	0.002	0.58	0.001
Ehhadh	sp| P07896| ECHP_RAT	0.56	0.005	1.76	0.001
Acsl1	sp| P18163| ACSL1_RAT	1.88	0.001	0.62	0.004
Ppox	tr| D3ZVN7| D3ZVN7_RAT	2.03	0.007	0.60	0.003

### Functional Classifications of DEPs

**FIGURE 4:**
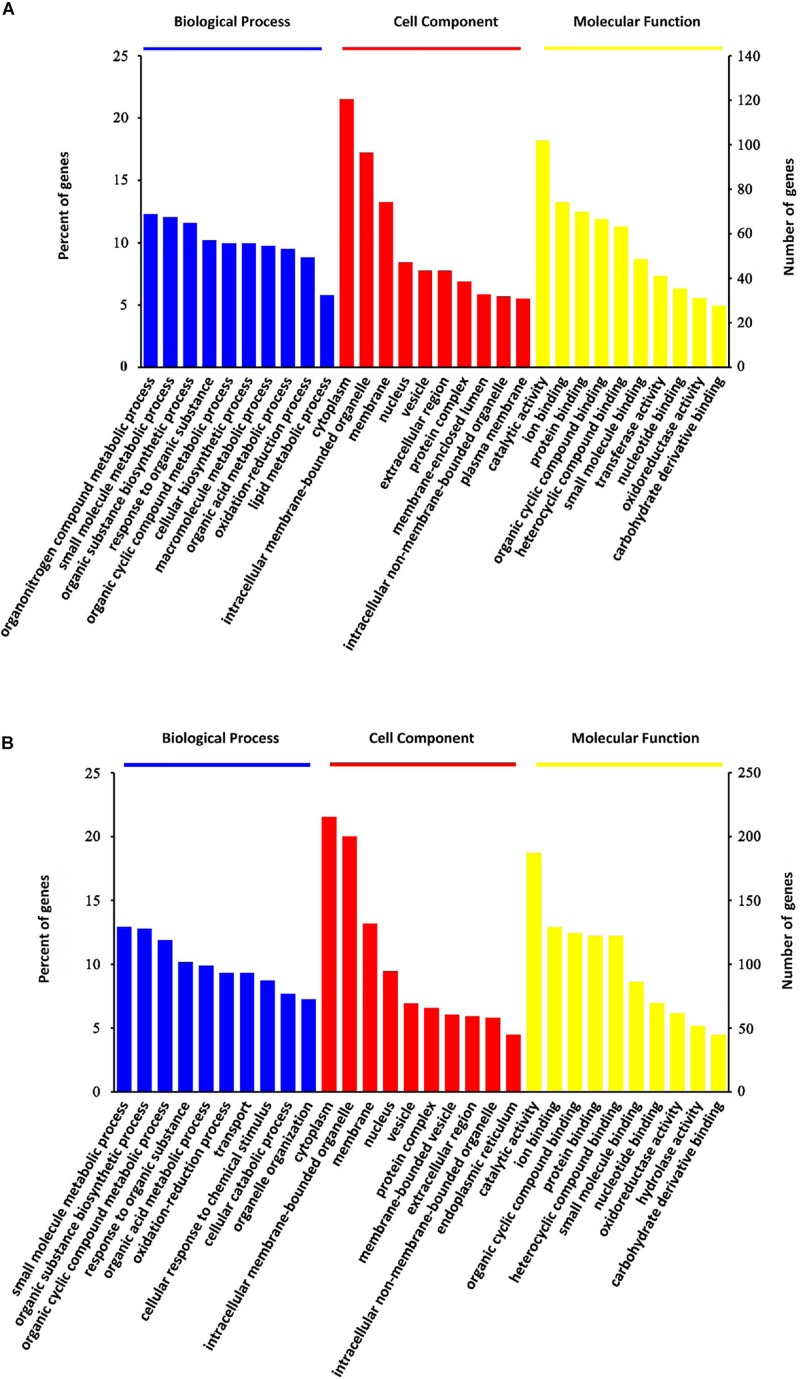
GO classification analysis. GO classification of steatosis-specific DEPs **(A)** and OMT-related DEPs **(B)** based on their biological process, cellular component and molecular function.

All these DEPs were analyzed by searching GO database to determine their participation in BP, CC and MF. As shown in Figure 4, the DEPs were mainly located in the cytoplasm, intracellular membrane-bounded organelle, membrane and nucleus. Their major MFs were catalytic activity, ion binding, protein binding, organic cyclic compound binding and heterocyclic compound binding. OMT-related DEPs were mainly involved in processes of small molecule metabolism, organic substance biosynthesis, organic cyclic compound metabolism, response to organic substance, organic acid metabolism, transport and oxidation-reduction.

### Pathway Enrichment and Protein-Protein Network Analysis

**FIGURE 5:**
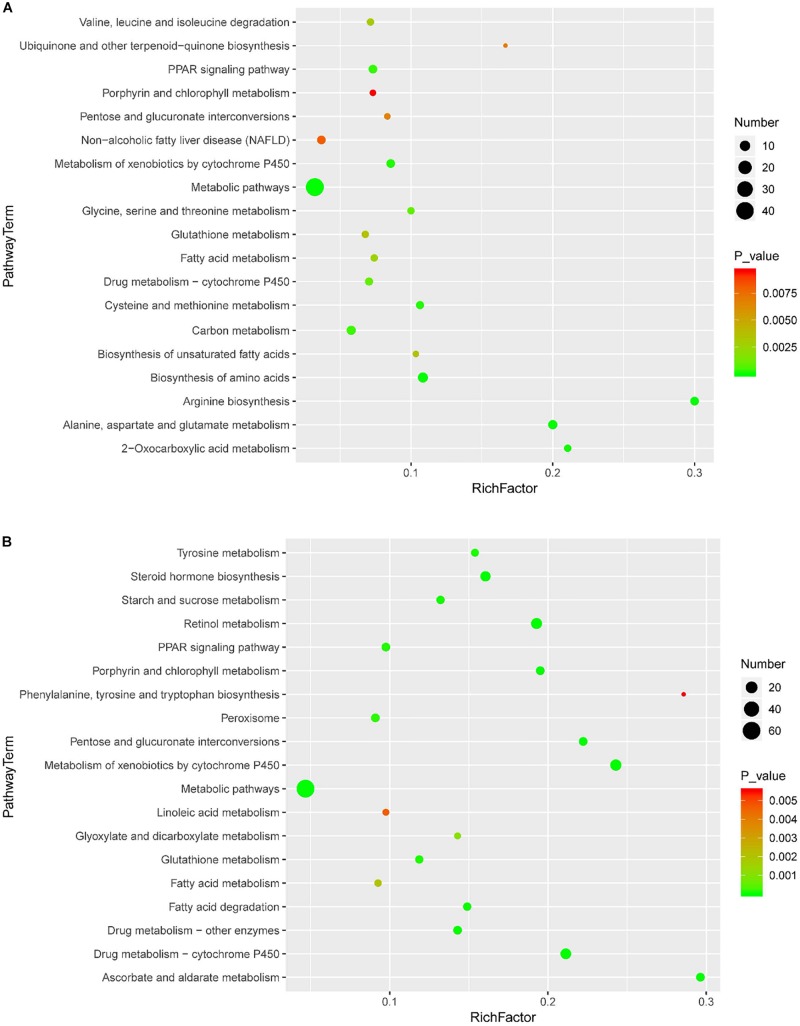
Scatter diagram of enriched KEGG pathways. Enriched KEGG pathways of steatosis-specific DEPs **(A)** and OMT-related DEPs **(B)**. Degree of enrichment was determined by the number of genes that enriched in one pathway, Rich factor and *P* value and. *Y*-axis represents the name of signaling pathway, *X*-axis represents the Rich factor. Point size means the number of differential expression genes in specific pathway, and the color of point means the range of *P* value.

**FIGURE 6:**
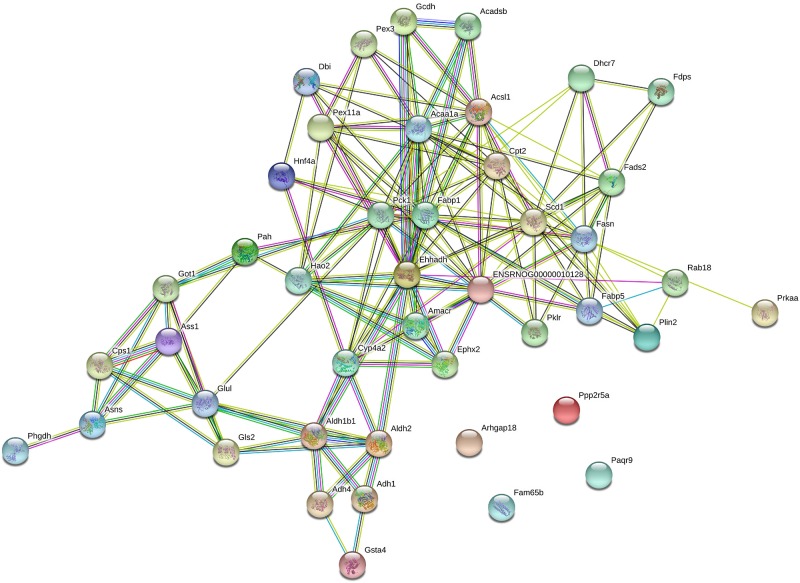
String network analysis of DEPs identified by iTRAQ-LC-MS/MS.

KEGG enrichment was performed to uncover the signal transduction pathways that DEPs may participate in. Steatosis-specific DEPs were mainly involved in carbon metabolism, biosynthesis of amino acids, arginine biosynthesis, cysteine and methionine metabolism, PPAR signaling pathway, glycine, serine and threonine metabolism, alanine, aspartate and glutamate metabolism, metabolism of xenobiotics by cytochrome P450, fatty acid metabolism and metabolic pathways. The top 10 signaling pathways that OMT-related DEPs participate in included tyrosine metabolism, steroid hormone biosynthesis, starch and sucrose metabolism, retinol metabolism, PPAR signaling pathway, peroxisome, glutathione metabolism, drug metabolism-cytochrome P450, fatty acid degradation and metabolic pathways (Figure 5). As shown in Figure 6, String network analysis identified various possible direct and indirect interactions among these DEPs. These proteins were mainly involved in fatty acid metabolism, fatty acid degradation, PPAR signaling pathway, peroxisome, non-alcoholic fatty liver disease, arginine biosynthesis, AMPK signaling pathway, biosynthesis of unsaturated fatty acids and biosynthesis of amino acids.

### Validation of Potential Therapeutic Targets by Western Blot Analysis

**FIGURE 7:**
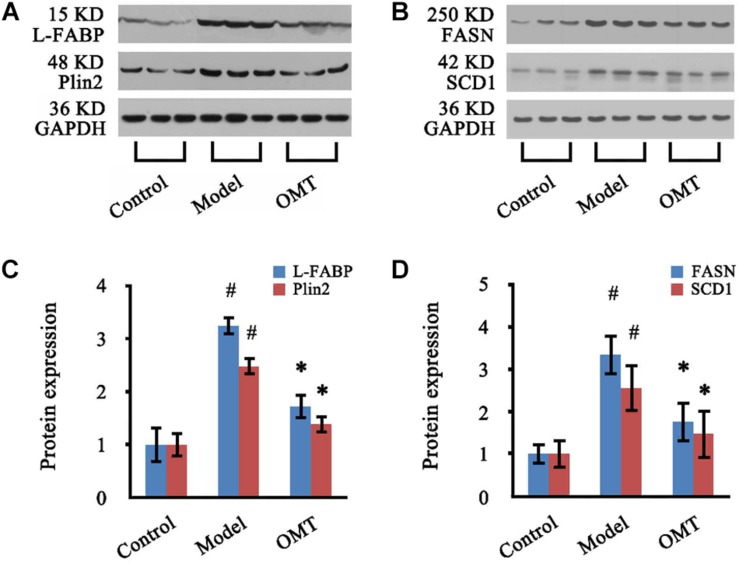
OMT decreases the expressions of L-FABP, Plin2, FASN and SCD1 in the liver of rats with steatosis. **(A,B)** Representative western blots of L-FABP, Plin2, FASN and SCD1 in liver tissues. GAPDH demonstrates the equal loading of proteins. **(C,D)** Graphic presentations show the expressions of L-FABP, Plin2, FASN and SCD1. The mean densities of these four proteins were normalized by that of GAPDH. The control samples were assigned a value of 1. Data are representative of 10 rats per group. #*P* < 0.001 vs. the control, ^∗^*P* < 0.001 vs. the model group.

Four significantly changed proteins: L-FABP, Plin2, FASN, and SCD1 were chosen for validation by western blot analysis. As shown in Figure 7, the expressions of L-FABP, Plin2, FASN, and SCD1 in the liver were significantly increased in the model group compared to the control and were 3.25-fold, 2.48-fold, 3.34-fold and 2.56-fold over the control, respectively. OMT treatment caused significant reductions in the levels of L-FABP, Plin2, FASN, and SCD1 by 52.92, 55.63, 52.69, and 57.42%, respectively. These results are concordant with our findings from iTRAQ analysis.

### OMT Increases Sirt1 Expression and AMPKα Phosphorylation

**FIGURE 8:**
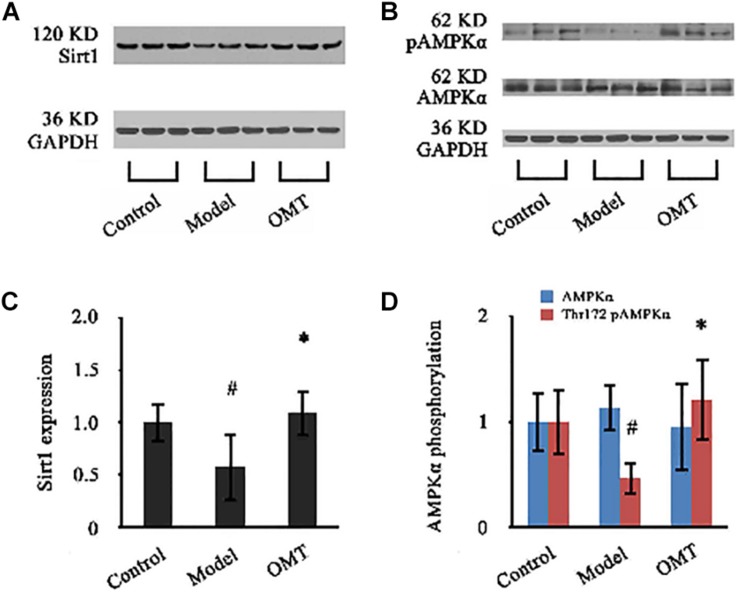
OMT increases Sirt1 expression and AMPKα phosphorylation. **(A,B)** Representative western blots of Sirt1, AMPKα and phospho-Thr172 AMPKα in liver tissues. GAPDH demonstrates the equal loading of proteins. **(C,D)** Graphic presentations show the expressions of Sirt1 and AMPKα and phosphorylation of Thr172 AMPKα. The mean densities of Sirt1 and AMPKα were normalized by that of GAPDH. AMPKα phosphorylation is represented as the relative ratio of the density of phospho-Thr172 AMPKα against that of total AMPKα. The control samples were assigned a value of 1. Data are representative of 10 rats per group. #*P* < 0.001 vs. the control, ^∗^*P* < 0.001 vs. the model group.

As shown in Figure 8, the expression of Sirt1 in the liver of the model rats was significantly reduced to 58.03% of the control. OMT caused a significant increase in Sirt1 expression up to 1.89-fold over the model group. No significant changes were detected in the protein expression of AMPKα among the three groups. However, Thr172 phosphorylation of AMPKα was significantly decreased compared to the control. In OMT-treated group, Thr172 phosphorylation of AMPKα was significantly increased and was 2.63-fold over the model group.

## Discussion

Current innovative strategies to treat NAFLD include identifying active ingredients of herbal extracts that can suppress lipid accumulation in the liver (Liu Q. et al., 2017; Zhang et al., 2018). The present study is not the first one reporting the anti-steatotic action of OMT. But importantly, this is for the first time displaying hepatic proteomic response to oxymatrine treatment in steatosis rats by iTRAQ analysis. Furthermore, our data have provided the novel evidence that OMT activates hepatic Sirt1/AMPK signaling which might be the potential therapeutic target of OMT for improving steatosis.

Although the precise mechanisms underlying the pathogenesis and progression of NAFLD still remain unclear, excessive lipid accumulation in the liver serves as a precursor for steatosis. It could stem from increased uptake of FFAs, elevated *de novo* lipogenesis and impaired fatty acid β oxidation. SREBP is a transcription activating factor of almost all genes that control the synthesis of fatty acids and TG in the liver. PPARα is another key regulator of the complex regulatory network of hepatic lipid metabolism (Cobbina and Akhlaghi, 2017). Therapeutic options targeting the improvement of lipid metabolism in the liver are crucial for the management of NAFLD. By creating HFHSD-induced steatosis rat model, we have provided strong support for OMT as a potential candidate for NAFLD treatment with high therapeutic efficiency. The body weight and liver weight of animals with steatosis were significantly reduced by OMT treatment. The sick greasy macroscopic appearances of the livers were remarkably improved. The serum levels of TG and TC as well as the hepatic TG and FFA levels were significantly decreased. Histopathological examination demonstrated that OMT effectively alleviated fatty degeneration in the liver. Moreover, OMT significantly attenuated lipid droplets (LDs) formation in oleic acid-induced steatotic hepatocytes and reduced intracellular TG and TC levels (see Supplementary Material).

Prior to the present study, limited literatures have documented the protective effect of OMT against hepatic steatosis (Shi L. et al., 2013). However, the starting point of these researches was based on the important roles of SREBF1 and PPARα in lipid synthesis and fatty acid β-oxidation, respectively. There were not any more details except the determination of the mRNA and protein expressions of SREBF1 and PPARα. A clear and detailed mechanism by which OMT improves steatosis remains largely unknown. In this study, by using iTRAQ-based proteomic method, a bunch of DEPs which up-regulate or down-regulate significantly by OMT treatment were identified. The data of GO analysis suggested that OMT-related DEPs were mainly located in the cytoplasm, intracellular membrane-bounded organelle, membrane and nucleus, participating mainly in the processes of small molecule metabolism, organic substance biosynthesis, organic cyclic compound metabolism, response to organic substance, organic acid metabolism, transport and oxidation-reduction. KEGG enrichment revealed that they were closely associated with following pathways: tyrosine metabolism, steroid hormone biosynthesis, starch and sucrose metabolism, retinol metabolism, PPAR signaling pathway, peroxisome, glutathione metabolism, drug metabolism-cytochrome P450, fatty acid degradation and metabolic pathways. Plin2, L-FABP, FASN, and SCD1 were selected as the most significantly differentially expressed targets.

Plin2 is constitutively located on the surface of LDs. It has been considered a reliable and sensitive marker for LDs and correlates positively with cytosolic TG content in hepatocytes. Plin2 plays a key role in fatty acid uptake, stabilization of LDs, lipid transport and storage. Plin2 regulated lipid exchange from LDs by facilitating direct protein-lipid interactions on the LDs surface (McIntosh et al., 2012). Study of clinical liver biopsy discovered that Plin2 mainly targeted inflamed ballooned hepatocytes. The frequency of Plin2-positive ballooned hepatocytes was correlated to inflammation and NAFLD activity score (Fujii et al., 2009). Suppression of Plin2 expression by global or liver-specific ablation of *plin2* gene resulted in decreased hepatic lipid accumulation and protected against diet-induced liver steatosis, inflammation and fibrosis (McManaman et al., 2013; Najt et al., 2016). Furthermore, whole-body loss of Plin2 exerts a protective effect in animals exposed long-term Western diet in part by suppressing hepatic SRBEP-1 and SRBEP-2 activity (Libby et al., 2016).

L-FABP is a small 14 kDa soluble protein and abundantly expressed in hepatocytes in high concentration, accounting for 2–5% of all soluble cytosolic proteins. L-FABP functions as a transporter of fatty acid in the cytoplasm. It mediates the transport of long-chain fatty acids (LCFAs) and other lipid ligands from cytoplasm to various organelles, such as nucleus, LDs, mitochondria, peroxisome and endoplasmic reticulum (Wang et al., 2015). Ablation of *L-FABP* gene impaired the ability of the liver to efficiently import and transfer fatty acids into glycerolipid biosynthesis resulting in a reduction of hepatic TG accumulation, and protected against diet-induced obesity and hepatic steatosis (Newberry et al., 2006; Martin et al., 2009, 2017; Mukai et al., 2017). Studies discovered that L-FABP and PPARα colocalized in the nucleus. L-FABP may serve to shuttle LCFAs into the nucleus for donating the ligands to PPARα and interacts directly with PPARα to influence transcriptional activity (Wolfrum et al., 2001; Huang et al., 2004). FASN and SCD1 are important lipogenesis-associated enzymes. FASN catalyzes the last step in *de novo* fatty acid synthesis. SREBP1c is the major transcriptional factor that regulates the expressions of FASN and SCD1 (Angeles and Hudkins, 2016; Zhang et al., 2017). FASN also controls the activation of PPARα under nutrient-deficient conditions to promote the adaptive response to fasting (Jensen-Urstad and Semenkovich, 2012).

Considering that OMT-related DEPs display a wide range of functions including fatty acid uptake, synthesis, transport, storage and degradation and participating PPAR signaling pathway, we speculate that the therapeutic target of OMT may locate on the upstream of the network of lipid metabolism regulation. Available studies have revealed that sirtuin 1 (Sirt1)/adenosine monophosphate-activated protein kinase (AMPK) signaling plays a pivotal role in lipid metabolism in the liver. AMPK, which serves as a sensor of cellular energy status, is a heterotrimeric enzyme that is composed by AMPKα (a catalytic subunit), AMPKβ (a scaffolding subunit) and AMPKγ (a regulatory subunit) (Day et al., 2017). AMPK activation by phosphorylation of Thr172 in α subunit decreases SREBP-1c expression to suppress lipid biosynthesis and activates PPARα to promote fatty acid β-oxidation (Smith et al., 2016). Sirt1, an NAD + -dependent protein deacetylase, is a critical regulator of AMPK activity in controlling hepatocellular lipid metabolism (Liou et al., 2018). Sirt1 deacetylated and inhibited the activity of SREBP-1c (Ponugoti et al., 2010). Sirt1-deficient mice lacked AMPK activity and had increased SREBP-1c expression that triggered hepatic steatosis and obesity (Zhang et al., 2016). Hepatocyte-specific loss of Sirt1 was shown to cause PPARα signal failure and a decrease in fatty acid β-oxidation (Purushotham et al., 2009). Our data indicated that OMT significantly increased hepatic expression of Sirt1, which is down-regulated in the liver of steatosis rats. Although the expression of total AMPKα remained unchanged, Thr172 phosphorylation of AMPKα was significantly increased following OMT treatment. The data have been further verified by *in vitro* experiment (see Supplementary Material). Thus, OMT may activate Sirt1/AMPK signaling to control the downstream key regulators of lipid synthesis, transport and degradation.

In summary, the present study has provided the evidence to confirm the efficacy of OMT on treating hepatic steatosis. A list of DEPs in the liver of steatosis rats by OMT treatment has been identified. Plin2, L-FABP, FASN, and SCD1 are considered the most significantly DEPs. Our data suggest a strong link between OMT and the BP of fatty acid uptake, synthesis, transport, storage, and degradation. Importantly, OMT activates the upstream Sirt1/AMPK signaling in the lipid metabolism regulatory network in the liver. Further studies are required to unravel the interaction, activity regulation, possible complexes and substrates of these proteins, and how they “cross-talk” with PPARα signaling pathway.

## Data Availability Statement

All datasets generated for this study are included in the article/Supplementary Material.

## Ethics Statement

All animals received humane care with strict accordance to the criteria outlined in the Guide for the Care and Use of Laboratory Animals. This study was reviewed and approved by the Committee on Animal Research and Ethics of Zhejiang Chinese Medical University (number ZSLL-2014-36).

## Author Contributions

HX, G-FC, Y-SM, H-WZ, and DF designed the study. HX, G-FC, Y-SM, G-HL, D-YC, JP, and DF, performed the proteomic analysis and western blotting. HX, G-FC, YZ, Y-HL, XM, and DF performed the animal experiments. HX, G-FC, H-WZ, and DF performed the statistical analyses, interpreted the data, and wrote the manuscript. HX, H-WZ, and DF contributed study materials and consumables. HX, YZ, and DF were responsible for troubleshooting of the experiments, editing of the manuscript, and the financial support. HX and G-FC contributed equally to this work. All authors contributed to the final version of the manuscript and approved the final manuscript.

## Conflict of Interest

The authors declare that the research was conducted in the absence of any commercial or financial relationships that could be construed as a potential conflict of interest.

## Footnotes

1 http://string-db.org

2 http://geneontology.org/

3 http://www.genome.jp/kegg/mapper.html

## Abbreviations

ALT: alanine aminotransferase
AMPK: adenosine monophosphate- activated protein kinase
AST: aspartate aminotransferase
BP: biological processes
CC: cellular component
DEPs: differentially expressed proteins
FASN: fatty acid synthase
FDR: false discovery rate
FFA: free fatty acid
HFHSD: high fat and high sucrose diet
GO: Gene Ontology
IAA: iodoacetamide
iTRAQ: isobaric tags for relative and absolute quantification
KEGG: Kyoto Encyclopedia of Genes and Genomes
LCFAs: long-chain fatty acids
L-FABP: liver fatty acid-binding protein1
LXR: liver-X-receptor
MF: molecular function
NAFLD: non-alcoholic fatty liver disease
OMT: oxymatrine
Plin2: perilipin2
PPAR α: peroxisome proliferator activated receptor alpha
SCD1: stearoyl-CoA desaturase 1
Sirt1: sirtuin 1
SREBF1: sterol regulatory element binding transcription factor 1
SREBP: steroid regulatory element binding protein
TC: total cholesterol
TG: triglyceride.
