# Supplementary material for: Hepatic Proteomic Changes and Sirt1/AMPK Signaling Activation by Oxymatrine Treatment in Rats With Non-alcoholic Steatosis
Source: Front Pharmacol. 2020 Mar 10;11:216. doi: 10.3389/fphar.2020.00216 (PMC7076077; doi:10.3389/fphar.2020.00216)
Supplement: Supplementary file 1 [file Data_Sheet_1.PDF]

Supplementary data

Hepatocytes and methods

Primary hepatocytes were isolated from male Sprague-Dawley rats (weighing 200-300g) by collagenase perfusion *in situ* and purified by Percoll centrifugation. Cells were cultured in DMEM containing10% fetal bovine serum. Cells were pretreated with OMT at a series of concentrations for 12h, then incubated with 0.5 mM oleic acid (OA) for 48h. MTT assay was performed to evaluate cell viability. 100μg/ml of OMT was screened as the suitable and effective concentration. Lipid droplets formation in cells was assayed by Oil Red O staining. The levels of TG and TC were determined by commercially available assay kits. Expressions of FASN, SCD1 and Sirt1 and phosphoryation of AMPKα were detected by western blotting.

Results

There were a large number of lipid droplets in primary hepatocytes incubated with OA, while pretreatment with OMT dramatically reduced lipid droplets formation (Fig. 1A). OA caused 4.08-fold and 4.23-fold increases in TG and TC levels, respectively. OMT pretreatment significantly decreased intracellular TG and TC levels (Fig. 1B-C).

As shown in Fig. 2, the expressions of FASN and SCD1 were significantly increased in OA-induced steatotic hepatocytes compared to

the control and were 2.98-fold and 3.56-fold over the control, respectively. OMT pretreatment caused significant reductions in FASN and SCD1 expressions. Conversely, the expression of Sirt1 in steatotic hepatocytes was significantly decreased to 32.48% of the control. Although there was no significant difference in AMPKα expression among the three groups, Thr172 phosphorylation of AMPKα was significantly decreased in steatotic hepatocytes compared to the control. OMT significantly increased Sirt1 expression and Thr172 phosphorylation of AMPKα.

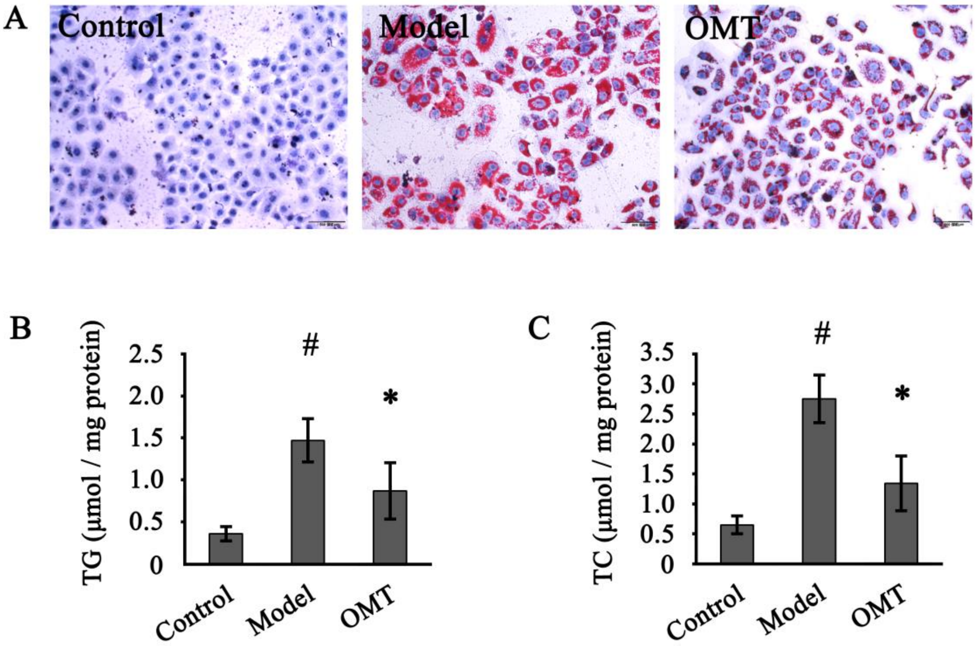

Fig. 1. OMT significantly reduces lipid accumulation in cultured steatotic hepatocytes. (A) Primary hepatocytes were either incubated with 0.5 mM OA at 37℃ for 48h to induce lipid accumulation or pretreated with 100μg/ml of OMT before OA stimulation. Lipid droplets formation was detected by Oil Red O staining. (B) Intracellular levels of TG and TC. Data are representative of three independent experiments. # $P < 0.001$ *versus* the control group, * $P < 0.001$ *versus* the model group.

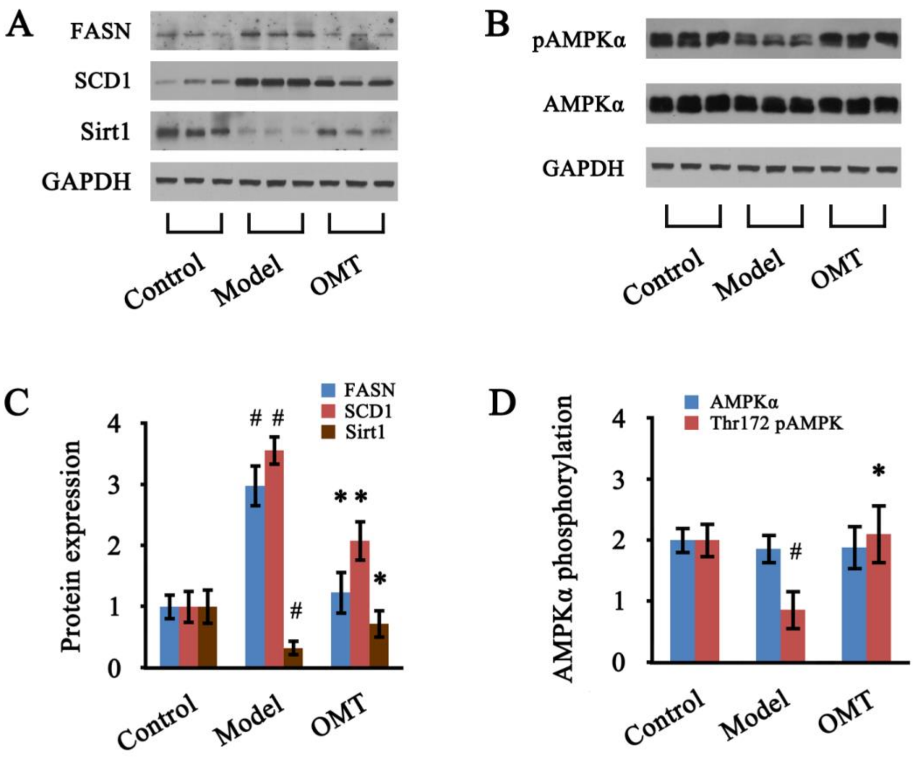

Fig. 2. OMT decreases the expressions of FASN and SCD1 and increases Sirt1 expression and AMPKα phosphorylation in OA-induced steatotic hepatocytes. (A, B) Representative western blots of FASN, SCD1, Sirt1, pAMPKα and AMPKα in cultured hepatocytes. GAPDH demonstrates the equal loading of proteins. (C, D) Graphic presentations show the expressions of FASN, SCD1, Sirt1, pAMPKα and AMPKα. The mean densities of FASN, SCD1, Sirt1 and AMPKα were normalized by that of GAPDH. AMPKα phosphorylation is represented as the relative ratio of the density of phospho-Thr172 AMPKα against that of total AMPKα. The control samples were assigned a value of 1. Data are representative of three independent experiments. # P <0.001 *versus* the control, * P <0.001 *versus* the model group.
